# Monocyte/macrophage-mediated venous thrombus resolution

**DOI:** 10.3389/fimmu.2024.1429523

**Published:** 2024-07-19

**Authors:** Meng-Jiao Lu, Jia-Qi Zhang, Zhou-Yu Nie, Tian-Hua Yan, Yong-Bing Cao, Li-Chao Zhang, Ling Li

**Affiliations:** ^1^ Institute of Vascular Disease, Shanghai TCM- Integrated Hospital, Shanghai University of Traditional Chinese Medicine, Shanghai, China; ^2^ Department of Physiology and Pharmacology, China Pharmaceutic University, Nanjing, China; ^3^ Department of Pharmacy, Shanghai Municipal Hospital of Traditional Chinese Medicine, Shanghai University of Traditional Chinese Medicine, Shanghai, China

**Keywords:** venous thromboembolism, thrombus resolution, macrophages, monocytes, inflammation, neovascularization

## Abstract

Venous thromboembolism (VTE) poses a notable risk of morbidity and mortality. The natural resolution of the venous thrombus might be a potential alternative treatment strategy for VTE. Monocytes/macrophages merge as pivotal cell types in the gradual resolution of the thrombus. In this review, the vital role of macrophages in inducing inflammatory response, augmenting neovascularization, and facilitating the degradation of fibrin and collagen during thrombus resolution was described. The two phenotypes of macrophages involved in thrombus resolution and their dual functions were discussed. Macrophages expressing various factors, including cytokines and their receptors, adhesion molecules, chemokine receptors, vascular endothelial growth factor receptors, profibrinolytic- or antifibrinolytic-related enzymes, and other elements, are explored for their potential to promote or attenuate thrombus resolution. Furthermore, this review provides a comprehensive summary of new and promising therapeutic candidate drugs associated with monocytes/macrophages that have been demonstrated to promote or impair thrombus resolution. However, further clinical trials are essential to validate their efficacy in VTE therapy.

## Introduction

Venous thromboembolism (VTE) comprising deep vein thrombosis (DVT) and its potential sequelae, including pulmonary embolism (PE) and post-thrombotic syndrome (PTS), is the third most common cardiovascular disease after myocardial infarction and stroke (1). The estimated VTE rate hovers approximately one to two individuals per 1,000 persons per year in Europe and the USA and is lower in Asia (2). The incidence of maternal VTE in China was estimated to be 0.13% (95% CI, 0.11–0.16), associated with cesarean section and advanced age (3). Moreover, approximately 15% of patients die within 1 year after being diagnosed with VTE, and survivors often suffer from various complications (4). Current treatments for VTE include anticoagulation therapy, thrombolytic therapy, surgical thrombectomy, inferior vena cava (IVC) filter placement, and others (5). However, these treatments have some drawbacks, such as increasing the risk of bleeding, being limited to fresh thrombus, not eliminating the occurrence of PTS, the absence of acceleration in thrombus resolution, etc. (6). Therefore, exploring new therapeutic strategies is essential for preventing and treating VTE.

Recently, much attention has been drawn to promoting the natural resolution of the venous thrombus as a potential alternative treatment strategy for VTE (7). It was known that the process of thrombus resolution involves a complex interaction between the venous endothelial cells, platelets, and innate immune cells such as neutrophils, monocytes/macrophages (Mo/MΦ), T cells, and mast cells (8). Among these cells, monocytes/macrophages were found to play a vital role in the dissolution and recanalization of blood clots through secreting a variety of factors rather than being essential for thrombogenesis (9, 10). To fully understand the function of Mo/MΦ in thrombus resolution, intrathrombotic monocytes/macrophages are being extensively studied (11, 12).

Therefore, the roles, major sources, and the two phenotypes of macrophages in VTE were described. Moreover, various factors expressed by macrophages, which could augment or repress the resolution of the venous thrombus, were outlined. Furthermore, the potential candidate drugs, which could promote or impair thrombus resolution and were associated with macrophage, were summarized. The novel strategies targeting Mo/MΦ may be promising for improving the treatment of VTE.

## Monocyte/macrophage recruitment and accumulation in the thrombus during thrombus resolution

The process of venous thrombosis (VT) is similar to local wound healing, with an early influx of neutrophils (PMN) into the thrombus, followed by Mo/MΦ (13). Recently, thrombus formation has been confirmed as an inalienable part of innate immunity, termed immunothrombosis (14, 15). Uncontrolled abnormal immune thrombosis causes severe damage, leading to thromboinflammation (14, 16). It is recognized that not only inflammation stimulates thrombosis but also thrombosis can in turn directly trigger inflammation, and a close, bidirectional relationship exists between inflammation and thrombosis (15, 17). A key link between inflammation and thrombosis is the formation of neutrophil extracellular traps (NETs) released by activated neutrophils, which act as scaffolds for the aggregation of erythrocytes and platelets (11, 18–21). Moreover, the activation of inflammasomes in neutrophils and the release of downstream proinflammatory cytokines IL-1β and IL-18 can enhance the recruitment and activation of platelets and monocytes (22–24). Inflammasome activation also promotes thromboinflammation by inducing the release of tissue factors by monocytes and macrophages to initiate the intrinsic pathway (22, 25). Furthermore, the activated platelets were found to not only play vital roles in thrombosis and hemostasis but also mediate inflammation through direct interactions with neutrophils as well as monocytes/macrophages in VTE (11, 18).

Up to now, monocytes and macrophages have also been shown to possess the ability to form extracellular traps which were named METs (26–28). METs released from human blood monocytes before differentiating into macrophages have an important host defense function which inhibits growth and dissemination of the human pathogenic yeast *Candida albicans* (29). METs were formed by macrophages exposed to the respiratory pathogen *Mannheimia haemolytica* and its leukotoxin (30). The death of macrophages with METs is called METosis, which is similar to NETosis (31). It was demonstrated that macrophage polarization may affect METosis and the M1-activated state is more prone to METosis after interaction with NETs (31, 32). Altogether, the web structures may be important for later immune cell responses and direct how the VT resolves (11). Many important functions of NETs or macrophage/monocyte extracellular traps in thrombus formation and resolution remain unclear.

Mo/MΦ are the primary effectors of immune-directed VT resolution (11). A previous study has shown that during venous thrombus resolution, the percentage area of thrombus covered by monocytes steadily increased in both human venous thrombi and experimental stenosis rat models (33). Monocytes initially appeared around the edge of the thrombus and gradually distributed more evenly as the resolution progressed. Moreover, monocyte content in the thrombus increased at 25 days than at 2 days after monocytes were injected intravenously into rats before thrombus induction (33). In short, these results indicated that both endogenous and exogenous supplementation of monocytes might migrate and accumulate in the thrombus during natural resolution.

It was demonstrated that the increase of macrophage numbers or monocyte recruitment into the thrombus could improve VT resolution and recanalization (34). The number of macrophages was shown to start increasing and peaking at 7 days after IVC ligation and then gradually decreasing until the experimental deadline of 21 days (35). Similarly, it was reported that the macrophage content in the thrombus of mice was the highest on day 14 compared to day 1 and day 28 after IVC stenosis (36). Flow cytometry analysis also showed that the proportion of CD45^+^CD11b^+^Gr-1^−^ monocytes in the thrombus reached its peak on day 10 after IVC stenosis and declined after that (37). Moreover, substantial macrophages, heterogeneously distributed along the length of the thrombi, were found to be infiltrated into the murine thigh and jugular VT induced by FeCl_3_ on day 4, and the intensity of macrophages in the thrombus on day 4 was correlated with the reduction of thrombus length and area from day 4 to day 6 (38).

Taken together, the number of monocytes/macrophages recruited and infiltrated in the thrombus increases throughout thrombus resolution, with a peak at the early and mid-term stages and then gradually decreasing with thrombolysis. Thrombotic macrophage content could predict the subsequent extent of DVT resolution *in vivo.*

## Major sources of monocytes/macrophages in the thrombus

Previous studies have implied that the majority of infiltrating monocytes in thrombi primarily originated from the circulation rather than resident cells in surrounding tissues (39).

Impaired thrombus resolution in *uPA* ^−/−^ mice transplanted with *uPA* ^−/−^ bone marrow was rescued by transplantation with WT bone marrow and cells expressing LacZ from the donor bone marrow presented in the thrombus after transplantation, indicating that the cells recruited into the thrombus were derived from the bone marrow (36). Later, Obi et al. found that the principal source of interleukin-6 (IL-6) in the thrombus was anti-inflammatory Ly6C^Low^ Mo/MΦ (a subpopulation of Ly6C^+^ Mo/MΦ) and the impaired VT resolution was reversed by adoptive transfer of bone marrow-derived monocytes (BMDMs) from WT mice into *IL-6* ^−/−^ mice, indirectly indicating that monocytes derived from BMDM might play an important role in the middle stage of thrombus resolution (40).

Furthermore, Kimball et al. found that impaired VT resolution could be restored by the adoptive transfer of anti-inflammatory CD11b^+^Ly6C^Lo^ Mo/MΦs from the blood and spleen, indicating that circulating CD11b^+^Ly6C^Lo^ Mo/MΦs were vital for normal VT resolution (10). Moreover, bone marrow-derived endothelial progenitor cells were found to be recruited into the thrombus during resolution and promoted neovascularization (41). The cells expressing a mixed macrophage and endothelial phenotype might represent a population of plastic stem cells that play a part in orchestrating thrombus recanalization.

Taken together, the origin of intrathrombotic Mo/MΦ seems to be from the bone marrow or circulation. However, the dedication of tissue-resident macrophages or cells derived from the “splenic reservoir” in dissolving the thrombus is still unclear.

## Phenotypic changes of macrophages during thrombus formation and resolution

Currently, accumulative studies have found the vital role of macrophage phenotypes in thrombus resolution and vein wall remodeling. According to cell surface antigens, macrophages are divided into two phenotypes: classically activated or proinflammatory (M1 type or M1-like) macrophages with CD11b^+^Ly6C^High^, CCR2^2+^, and CX3CR1^+^ antigen expression; and alternatively activated or anti-inflammatory macrophages (M2 type or M2-like) with CD11b^+^Ly6C^Low^, CCR2^−^, and CX3CR1^2+^ antigen expression. M1-type macrophages express a defined set of proinflammatory cytokines such as IL-1β, IL-6, IL-12, inducible nitric oxide synthase (iNOS), and CCR2 (42). However, M2 type macrophages express IL-10, arginase 1 (Arg-1), and CD206, with profibrinolytic and inflammation-resolving activity (43).

An initial study showed that M2-like macrophages predominantly in the experimental VT model might impair thrombus resolution (44). However, later research demonstrated that infiltrating monocyte/macrophages derived from the blood could differentiate into inflammatory (CD11b^+^Ly6C^High^) or pro-resolving (CD11b^+^Ly6C^Low^) subtypes and the pro-resolving CD11b^+^Ly6C^Low^ Mo/MΦs were vital for normal VT resolution (10). Recently, it was found that the inflammatory Ly6C^High^ monocytes could enhance thrombus formation, and reducing inflammatory monocyte numbers could inhibit established thrombus growth and promote resolution (37, 45).

Moreover, current studies have shown that macrophage polarization can cause changes in macrophage function, including different migratory behaviors or alterations in extracellular matrix remodeling (46–48). It was reported that proinflammatory polarization of macrophages could boost NET degradation through enhanced macropinocytosis and inhibition of macropinocytosis led to increased thrombus NET burden and reduced thrombus resolution in mice with IVC stenosis (49).

**Figure 1:**
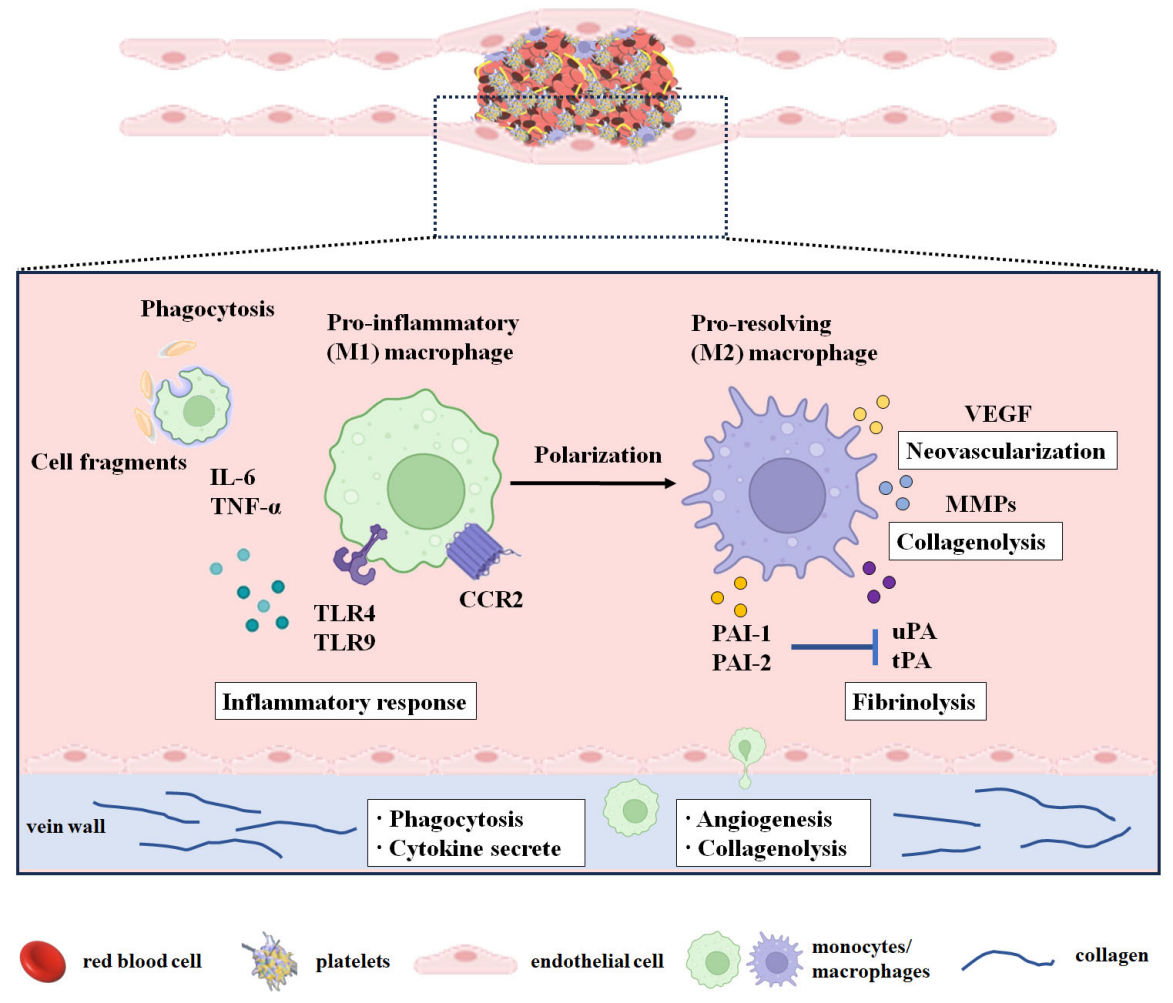
Monocytes/macrophages in the resolution of the venous thrombus. The period from day 1 to day 3 after modeling or thrombosis was considered the thrombus formation period. The early stage of thrombus resolution was considered from day 4 to day 7 after thrombosis. The middle and late stages of thrombus resolution were considered from day 8 after thrombosis. During thrombus resolution, macrophages transform from M1 type to M2 type and participate in thrombus resolution by clearing necrotic cells and matrix debris and promoting neovascularization, profibrinolysis, and collagenolysis. M1-type macrophages infiltrate into the thrombus during thrombus formation and play a major role in the early stage of thrombus resolution, while M2-type macrophages play a vital role in the middle and late stages of thrombus resolution. CCR2, C-C chemokine receptor 2; IL-6, interleukin-6; MMPs, matrix metalloproteinases; PAI-1, plasminogen activator inhibitor-1; PAI-2, plasminogen activator inhibitor-2; PECAM-1, platelet endothelial cell adhesion molecule 1; TNF-α, tumor necrosis factor-α; TNF-Rp55, tumor necrosis factor receptor p55; TLR4, Toll-like receptor 4; TLR9, Toll-like receptor 9; tPA, tissue-type plasminogen activator; uPA, urokinase-type plasminogen activator; VEGF, vascular endothelial growth factor.

Taken together, M1 macrophages mainly enhance thrombus formation, and M2-type macrophages mainly promote thrombus resolution (**Figure 1**). Therefore, altering the M1/M2 macrophage balance may accelerate thrombus resolution and allow the development of novel therapies to treat venous thrombus and to prevent PTS.

## Monocytes/macrophages augment neovascularization, profibrinolysis, and collagenolysis during thrombus resolution

Mo/MΦ are multifunctional leukocytes concerned with VT resolution. These leukocytes are involved in clearing necrotic cells and matrix debris, promoting neovascularization and profibrinolysis, and degrading the extracellular matrix (**Figure 1**).

### Monocytes/macrophages promote neovascularization

Thrombus neovascularization has been demonstrated to be another key event during thrombus resolution and recanalization. Macrophages predominate in the mid and later stages of resolution and are likely to be the most prominent effector cells in this procedure. Intrathrombotic macrophages express and release proangiogenic factors, chemokines, and cytokines, such as IL-8, VEGF, basic fibroblast growth factor (bFGF), and placental growth factor (PLGF), which stimulate capillary formation and neovascularization and modulate the recruitment of immune cells including monocytes/macrophages (50, 51).

VEGF is a growth factor with potent proangiogenic activity (52). VEGF concentration in the thrombus was found to be increased between 1 day and 7 days, and VEGF was distributed in monocytes/macrophages, endothelial cells, and spindle-shaped cells in the thrombus (53, 54). Similarly, adenovirus VEGF-transfected macrophage injection promoted thrombus resolution and enhanced vein lumen recanalization (55). Moreover, the antiangiogenic drug 2-methoxyestradiol attenuated the resolution of the venous thrombus, accompanied by a decrease in VEGF and PLGF levels, as well as neutrophil and macrophage contents in the vein of thrombosis (56). Hence, the potential prothrombotic effects of antiangiogenic drugs ought to be carefully thought over when treating patients with VTE.

bFGF, a growth factor known for its potent stimulation of cell proliferation, differentiation, growth, survival, and angiogenesis during development, has recently garnered attention for its therapeutic potential in wound healing, cardiovascular disease, and nervous system disorders (57, 58). bFGF content in the thrombus was found to be positively linearly correlated with time and gradually increased by more than 300-fold on day 28 in a stenosis rat model. Interestingly, the bFGF content in the adjacent vena cava wall and the serum bFGF level remained unchanged over time. The bFGF was found to be expressed in mononuclear cells, spindle-shaped cells, and extracellular matrix (53). These results implied that bFGF expressed, at least partially by monocytes, in organizing the thrombus might be involved in thrombus resolution. However, the role and potential mechanism of bFGF in thrombus resolution need further research.

Taken together, monocytes/macrophages play a pivotal role by expressing and releasing factors such as angiogenic growth factors, cytokines, and chemokines to enhance intrathrombotic neovascularization, which conversely recruit and activate monocytes/macrophages during thrombus resolution.

### The phagocytosis and proteolysis of monocytes/macrophages

It was demonstrated that intrathrombotic macrophages could engulf necrotic tissue, clear cellular debris, and release proteolytic enzymes such as matrix metalloproteinases-2 (MMP-2), matrix metalloproteinases-9 (MMP-9), urokinase-type plasminogen activator (uPA), and tissue-type plasminogen activator (tPA), to dissolve the surrounding matrix and fibrin, thereby promoting thrombolysis and recanalization (54, 59).

It was demonstrated that NETs could be degraded by macrophages via phagocytosis (32, 60, 61). The blocking of macropinocytosis in the stenosis mice model inhibited thrombus resolution and increased the NET content in the thrombus (49). F4/80 (a macrophage marker) was found to be co-localized with MMP-2, MMP-9, and uPA in the thrombus (59). Macrophages transfected with adenovirus uPA (ad-uPA) were shown to raise fibrinolytic activity, and upregulating uPA by systemic administration of transfected cells could promote thrombosis resolution in mice (62). In brief, macrophages promote thrombus resolution through phagocytosis or by releasing a pro-fibrinolysis enzyme (uPA) or matrix-degrading enzymes (MMP-2 and MMP-9).

## Various factors associated with monocytes/macrophages affect thrombus resolution

It is worth noting that several inflammatory factors or their receptors [IL-6, interferon-γ (IFN-γ), tumor necrosis factor receptor p55 (TNF-Rp55α), Toll-like receptor 4 (TLR4), TLR9], chemokine receptor (CCR2), adhesion molecule (platelet endothelial cell adhesion molecule 1 (PECAM-1)], VEGFR2, plasmins [uPA, tPA, plasminogen activator inhibitor type 1 (PAI-1), plasminogen activator inhibitor type 2 (PAI-2), MMP-2, MMP-9], and one gene (P53) are involved in thrombus resolution and associated with monocytes/macrophages (**Table 1**).

**Table 1:** Various factors associated with monocytes/macrophages affecting thrombus resolution.

Category	Various factor	Gene deletion	DVT model	Thrombus resolution status	Mo/MΦ content in thrombus	Mechanisms	References
Cytokine or its receptor	IL-6	Gene deletion	Stenosis	Impaired	Decrease or no change	↑ Thrombus mass, collagen areas, fibronectin; ↓ blood flow recovery, neovascularization; adoptive transfer of WT BMDM reversed impaired thrombus resolution	(40, 59)
	IFN-γ	Gene deletion	Stasis	Promote	No change	↓ Thrombus size, collagen content, blood flow recovery; ↑ neovessels, MMP-9 and VEGF expression	(63)
	TNF-Rp55	Gene deletion	Stenosis	Impaired	No change	↓ Thrombus shrinking, blood flow recovery, uPA, MMP-2 and MMP-9	(64)
	TLR4	Gene deletion	Stasis	Impaired	Decrease	↑ Thrombus weight/length ratios, collagen content; ↓ neutrophil content, MCP-1, MMP-9, VEGF, IFNβ, and MCP-5 expression in the thrombus	(65)
	TLR9	Gene deletion	Stasis	Impaired	Decrease or increase	↑ Thrombus size and weight, Cit-H3, HMGB-1, and uric acid, αSMA, and fibronectin expression in the vein wall; ↓ thrombus collagen, neovascularization, free fibrinogen, CD31, VE-cadherin expression, IFN-α, IL-1α, IL-18, and IL-2 levels in the vein wall	(66, 67)
Chemokine receptor	CCR2	Gene deletion	Stasis	Impaired	Decrease	↑ Thrombus volume and collagen; ↓ neovascularization	(34, 68)
Adhesion molecule	PECAM-1/CD31	Gene deletion	Stenosis	Impaired	Decrease	↑ Thrombus volumes and lengths, cross-sectional area, collagen; ↓ vessel formation	(69)
Growth factor receptors	VEGFR-2/KDR/Flk-1	Endothelial cell-specific deletion	Stenosis	Impaired	Decrease	↑ Thrombus weights, cross-sectional areas and volumes of the thrombi; ↓ microvessel density	(70)
Plasmin	uPA	Gene deletion	Stasis	Unaffected (early stage)	Decrease	Unchanged thrombus sizes; ↑ IFN-γ, PAI-1, active MMP-2 and MMP-14; ↓ PMN, plasmin activity, collagen type IV and fibrinogen in the thrombus	(71)
	uPA	Gene deletion	Stenosis	Impaired (mid and late stages)	Decrease	↑ Thrombi size; transplanting WT bone marrow restores thrombus resolution in *uPA^−/−^* mice	(36)
	tPA	Gene deletion	Stenosis	Unaffected	No change	Unchanged thrombus sizes	(36)
	PAI-1	Gene deletion	Stasis	Promote	Increase	↓ Thrombus weight, MMP-2 and MMP-9 activities	(72)
	PAI-1	Overexpression	Stasis	Impaired	Decrease	↑ Thrombus weight, procollagen I and IIIα gene expression; ↓ vein wall collagen content, intimal fibrosis, MCP-1, MMP-2 and MMP-9 activity, and TIMP-1 level	(73)
	PAI-2	Gene deletion	Stasis	Promote	Decrease	↓ thrombus weight, PAI-1 levels; ↑ uPA activity, CXCL2 levels and neutrophil content	(72)
	MMP-2	Gene deletion	Stasis	Impaired (early stage)	Decrease	↑ Thrombi size; ↓ PAI-1 levels in the thrombus	(71)
	MMP-9	Gene deletion	Stasis	Impaired	Decrease	↑ Thrombus weights; ↓ vein wall compliance, extracellular matrix and collagen deposition	(74)
Others	p53	Gene deletion or myeloid-specific deletion	Stasis	Impaired	No change	↑ Thrombus weights, collagen deposition and IL-6 expression; ↓ MMP-2 expression; repressed macrophage polarization toward an M2-like phenotype	(75)

↑, increase or enhance; ↓, decrease or inhibit.

bFGF, basic fibroblast growth factor; BMDM, bone marrow-derived monocyte; Cit-H3, citrullinated histones; CCR2, C-C chemokine receptor 2; CXCL2, C-X-C motif ligand 2; DVT, deep vein thrombosis; HMGB1, high mobility group protein B1; IFN-γ, interferon-γ; IL-1β, interleukin-1β; IL-6, interleukin-6; MCP-1, monocyte chemotactic factor-1; MMP-2, matrix metalloproteinase-2; MMP-9, matrix metalloproteinase-9; Mo/MΦ, monocytes/macrophages; PAI-1, plasminogen activator inhibitor-1; PAI-2, plasminogen activator inhibitor-2; PECAM-1, platelet endothelial cell adhesion molecule 1; PMN, polymorphonuclear cell; α-SMA, alpha smooth muscle actin; TNF-α, tumor necrosis factor-α; TNF-Rp55, tumor necrosis factor receptor p55; TIMP-1, tissue inhibitor of metalloproteinase 1; TLR4, Toll-like receptor 4; TLR9, Toll-like receptor 9; tPA, tissue-type plasminogen activator; uPA, urokinase-type plasminogen activator; VEGF, vascular endothelial growth factor; WT, wild type.

### Cytokines and their receptors

#### Interleukin-6

IL-6 is a pleiotropic proinflammatory cytokine that is not only a key modulator in homeostasis and inflammation but also involved in the pathogenesis of various diseases (76). IL-6 in the thrombus was gradually increased after IVC ligation and mainly located in F4/80-positive macrophages. IL-6 derived from macrophages was involved in VT resolution and IL-6 deficiency delayed thrombus resolution (59). However, whether IL-6 promotes VT resolution through the Stat3 signaling pathway *in vivo* needs further research. Recently, Ly6C^low^ (CD11b^+^CD3^−^CD19^−^Ter119^−^NK1.1^−^) Mo/MΦ were further found to be the predominant leukocyte source of IL-6. Consistent with a previous study, IL-6 deficiency impaired VT resolution through dysregulation of MMP-9 (40). Therefore, enhancing monocyte IL-6 signaling may provide a potential target to improve thrombus resolution without affecting anticoagulant function.

#### Interleukin-1β

IL-1β is one of the most prominent inflammatory mediators leading to fever and immune activation by binding to IL-1 receptor 1 (77). A previous study reported that serum IL-1β level and IL-1β mRNA expression increased in thrombus tissue at 24 h in a stasis rat model (78). However, it was not shown which cells in the thrombus IL-1β originates from (78). It was found that IL-1β neutralizing antibodies attenuated inflammasome activation and reduced stasis-induced thrombosis under hypoxic conditions and IL-1β mRNA expression increased in peripheral blood mononuclear cells from patients with altitude-induced venous thrombosis, indicating that IL-1β played a compelling role in thrombus formation (79). Moreover, the Canakinumab Anti-inflammatory Thrombosis Outcome Study has demonstrated the beneficial effect of anti-inflammatory therapy targeting IL-1β on a recurrent cardiovascular event (22, 80). Additionally, venous thrombosis in CD39-deficient mice was reduced by IL-1β blockade with a neutralizing IL-1β antibody or with an inhibitor of the IL-1 receptor (81). Thus, the strategies for targeting the blockade of IL-1β in VTE should be considered in the future. However, the role of IL-1β during thrombus resolution has not been reported yet.

#### Interferon-γ

IFN-γ, a pleiotropic cytokine, serves as a central coordinator of the immune response with antiviral, antiproliferative, proapoptotic, antiangiogenic, antitumor, and immunomodulatory properties (82). IFN-γ is mainly produced by T cells, natural killer cells, macrophages, and mucosal epithelial cells (83).

Intrathrombotic IFN-γ levels were found to be gradually increased after IVC ligation, and IFN-γ expression was mainly distributed in F4/80-positive macrophages in the thrombus, indicating that IFN-γ in the thrombus was produced mainly by infiltrating macrophages. IFN-γ derived from macrophages was involved in VT resolution and IFN-γ deficiency enhanced thrombus resolution possibly through upregulating MMP-9 and VEGF expression (54). Therefore, IFN-γ may become a molecular target for developing new drugs to promote thrombus resolution in patients with VT. Nevertheless, the mechanism of IFN-γ produced by macrophages in dissolving blood clots needs further exploration.

#### Tumor necrosis factor receptor p55

TNF-α, a well-known proinflammatory cytokine, was mainly generated by activated macrophages, T lymphocytes, and natural killer cells. The biological functions of TNF-α are mediated by its two main receptors: type 1 receptors (TNFR1, also known as TNFRSF1A, CD120a, and p55) and type 2 receptors (TNFR2, also known as TNFRSF1B, CD120b, and p75) (84). The mRNA levels of TNF-α and TNF-Rp55 in the thrombus gradually increased after IVC ligation, and TNF-α and TNF-Rp55 were mainly expressed in F4/80-positive macrophages (85). The TNF-α–TNF-Rp55 axis might increase the expression of uPA, MMP-2, and MMP-9 in intrathrombotic macrophages, thus improving thrombus resolution in mice (64).

#### Toll-like receptor 4

TLR4, a member of the Toll-like receptor family, initiates the innate immunity response and mediates inflammatory responses by recognizing exogenous pathogen-associated (PAMPs) and endogenous danger-associated molecular patterns (DAMPs) (86). It was found that TLR4 deficiency impaired VT resolution, together with reduced neutrophil and macrophage infiltration into the thrombus and lower MCP-1, MMP-9, VEGF, IFNβ, and MCP-5 expression in the thrombus (65). However, the role of macrophages in TLR4 deficiency damaging thrombolysis needs further research.

#### Toll-like receptor 9

TLR9, an intracellular TLR, is located in endosomal compartments and is implicated in immunity, inflammation, and several autoimmune diseases (87).

TLR9^+^ cells were found to be distributed in the intraluminal tissue of human chronic post-thrombotic veins and co-localized with CD68-positive cells in the thrombus (66). TLR9 signaling in macrophages plays a vital role in later VT resolution and is related to necrosis clearance, without affecting later vein wall fibrosis (66). TLR9 deletion damaged early VT resolution, independent from MyD88 but partially dependent on the NOTCH ligand delta-like 4 (DLL4) (67). However, how TLR9 affects the PMN and macrophage influx into thrombus remains unclear.

### Chemokines and their receptors

#### Cysteine-cysteine chemokine receptor

Cysteine-cysteine (CC) chemokine receptor (CCR2), is the receptor for C-C chemokine ligand 2 (CCL2), also known as monocyte chemoattractant protein-1 (MCP1). CCR2 and its ligand CCL2 regulate the recruitment and activation of monocyte/macrophage chemotaxis in various inflammatory diseases (88, 89). CCR2 deletion was found to inhibit thrombus resolution and monocyte recruitment (34). Similarly, CCR2 deficiency impaired early thrombus resolution with fewer thrombus monocytes, partly due to reduced MMP-9 activity (68). In brief, CCR2 activation was important for the regulation of monocyte recruitment into the thrombus and represented a potential target for enhancing thrombus resolution.

#### Platelet endothelial cell adhesion molecule 1

PECAM-1, also known as CD31, is a 130-kDa transmembrane glycoprotein expressed by cells interacting at the blood vessel interface and functions as a cell adhesion molecule with proangiogenic and proinflammatory activities (90, 91). PECAM-1 is known to participate in leukocyte migration and angiogenesis, which are the critical parts of resolving the venous thrombus (92). PECAM-1 deficiency delayed venous thrombus resolution with less macrophage invasion, and plasma-soluble PECAM-1 might possess a predictive value for PTS after acute DVT (69). However, the data were limited by the relatively small sample size and the use of a PECAM-1 deficiency mice model compared to secondary PECAM-1 deficiency in humans. Therefore, the cellular sources of PECAM-1 and its predictive value are worthy of further study.

### Growth factors

#### VEGF-R2/kinase insert domain protein receptor

VEGF-R2/kinase insert domain protein receptor (VEGF-R2/KDR/Flk-1), a type III transmembrane kinase receptor, is predominantly expressed in vascular endothelial cells and plays a major role in angiogenesis (93). It was reported that VEGF-R2 and VEFGA expressions were lower in white chronic thromboembolic pulmonary hypertension (CTEPH) thrombi compared with those in organizing DVT and organizing thrombi from aortic aneurysms (70). Furthermore, VEGF-R2-specific deletion in endothelial cells was found to delay thrombus resolution with lowering macrophage counts probably through ablation of thrombus vascularization (70). Given that the VEGF-R2 gene in monocytes was not targeted by gene deletion, it was possible that angiogenesis might arise first, then allow monocytes to recruit into the thrombus during thrombus resolution.

### Enzymes related to fibrinolysis and collagen degradation

#### Tissue-type and urokinase-type plasminogen activators

tPA and uPA are serine proteases with a key role in catalyzing the conversion of the inactive zymogen plasminogen into activated protease plasmin, which degrade fibrin and multiple components of extracellular matrix (ECM) turnover and basement membrane, including collagen, vitronectin, laminin, fibronectin, and proteoglycans (94).

Previous studies have shown that both tPA and uPA activities in the thrombus were increased during thrombolysis and expressed by infiltrating monocytes (95, 96). Early thrombolysis was found to be independent of uPA and leukocyte infiltration but related to increased IFN-γ and MMP-14 levels and MMP-2 activity (71). The mid and late stages of VT resolution were modulated by uPA but were unaffected by tPA deletion (36). The effect of uPA deficiency on thrombus resolution was related to delayed recruitment of monocytes into the thrombus, and bone marrow-derived cells might play a vital role in thrombus resolution (36).

In a word, the resolution of the venous thrombus was dependent on uPA rather than tPA, and the effect of uPA promoting thrombus resolution may be associated with infiltrating monocytes in the thrombus.

#### Plasminogen activator inhibitor types 1 and 2

PAI-1, a member of the serine protease inhibitor (serpin) superfamily, is a key physiological inhibitor of both uPA and tPA. PAI-2, also known as serpinB2, originally identified as an inhibitor of uPA, is also a serine protease inhibitor that has the ability to inhibit many extracellular proteases, but it has a lower efficacy on tPA and uPA compared to PAI-1.

PAI-1 deficiency was found to not only stimulate thrombus resolution but also mitigate thrombus formation which is associated with the increase at the early stage and the decrease of MMP-2 and MMP-9 activity at the later stage (72). Moreover, macrophage percentage was also increased at the early stage isolated from whole clots with the vein wall (72). PAI-1 overexpression attenuated vein wall fibrosis after DVT, probably by decreasing macrophage infiltration (73).

PAI-2 deficiency was found to enhance thrombolysis without impacting thrombus formation, and this enhancement was linked to the increased uPA activity and decreased PAI-1 levels, without affecting MMP-2 and MMP-9 activities (72). Moreover, PAI-2 deficiency enhanced early neutrophil recruitment through elevating the neutrophil chemoattractant CXCL2 levels, with a decrease of macrophage number (72). The results indicated that PAI-2 might be a potential therapeutic target for accelerating thrombus resolution.

Taken together, there are not only similarities but also differences between PAI-1 and PAI-2 in VT formation and resolution.

#### Matrix metalloproteinase-2 and matrix metalloproteinase-9

MMP-2 and MMP-9 (also known as gelatinases), two members of MMPs belonging to the gelatinase family, degrade ECM. Their involvement spans various biological processes, including alterations in cell–cell and cell–ECM interactions, cleavage of cell surface proteins, and extracellular environment protein cleavage (97).

MMP-9 expression and activity in the vein wall and thrombus were elevated in mice on days 2 to 3 after IVC ligation. MMP-9 deletion impaired thrombus resolution and MMP-9 derived from bone marrow played a role in thrombus resolution which was linked to collagen deposition and macrophage recruitment (74).

However, early thrombus resolution was dependent on MMP-2, which affects intrathrombotic monocyte influx and collagen deposition (71). However, the role and mechanism of MMP-2 in intrathrombotic monocyte influx and activation in early thrombus resolution need further study.

In brief, gene deletion of both MMP-2 and MMP-9, which have collagenolytic and elastolytic activity, impeded thrombus resolution possibly through collagen deposition and monocyte/macrophage infiltration into the thrombus.

### Others

#### p53

The tumor suppressor p53, as a genome guardian, plays a vital role in cell cycle control, senescence, DNA repair, apoptosis, and cellular stress responses through a variety of transcriptional and non-transcriptional activities (98). Interestingly, global deletion of p53, or p53 deficiency in myeloid cells, or the p53 inhibitor pifithrin was found to damage thrombus resolution via repressing intrathrombotic macrophage polarization toward an M2-like phenotype (75).

#### Diet-induced type 2 diabetes mellitus

Diabetic mice received a high-fat diet containing 45% kcal of fat for 10 weeks. The diet-induced type 2 diabetes was found to impair DVT resolution through increasing macrophage content and altering inflammatory, fibrinolytic, and MMP responses (99).

## Potential candidate drugs associated with monocytes/macrophages affect thrombus resolution

Up to now, only a limited number of inflammatory factors, chemotactic factors, associated antibodies, or compounds, which correlate with monocytes/macrophages, have been shown to enhance or attenuate VT resolution (**Table 2**).

**Table 2:** Potential candidate drugs associated with monocytes/macrophages affecting thrombus resolution.

Category	Potential candidate drug	DVT model	Mo/MΦ content	Mechanisms	References
Promoted thrombus resolution					
Cytokines	Recombinant murine TNF-α	Stasis	No change	↓ Thrombus weights; ↑ blood flow recovery, without affecting coagulation functions; ↑ uPA, MMP-2, MMP-9 gene expression in macrophages derived from WT mice *in vitro*	(64)
	Recombinant murine IL-6	Stasis	No determined	↓ Thrombus mass; ↑ blood flow recovery	(59)
Chemokines	Recombinant human IL-8	Stasis	No determined	↑ Thrombus blood flow, absolute femoral venous pressure, PMN content, neovascularization, and fibrosis; ↓ VEGF level	(100)
	Recombinant rat MCP1/CCL2	Stenosis	Decrease in the vein wall or no change	↓ Thrombus area and specimen weight; ↑ organization scores	(34, 101)
Growth factor	Recombinant human VEGF	Stenosis	Increase in the center of the thrombus	↑ The area of lumen recanalization, organization score	(102)
Antibodies	Anti-factor XI antibody	Stenosis	Decrease in the thrombus and vein wall	↓ Thrombus weights and volumes, circulating monocytes; ↑ collagen deposition, CD31 and SMA expression	(103)
	Anti-IFN-γ antibody	Stasis	No change	↓ Thrombus mass and collagen areas; ↑ blood flow recovery; ↑ MMP-9 and VEGF mRNA expression	(63)
PAI-1 inhibitor	Tiplaxtinin	Stenosis	No change in the vein wall	↓ Intimal thickening and fibrosis scores; ↑ PMN extravasation in the vein wall	(104)
P-selectin inhibitor	PSI-421	Stasis	Not determined	↑ Percent vein reopening; ↓ vein wall inflammation	(105)
Colony-stimulating factor	rhG-CSF	Stenosis	Increase	↑ Organization rate and neovessels in thrombi, monocytes in peripheral blood	(106)
PHD inhibitor	L-mimosine	Stenosis	Increase	↓ Thrombus weight and volume; ↑ vein recanalization, neovascularization; ↑ HIF1α, VEGF, and VEGFR expression in the thrombus and neutrophil number in the thrombus and vein wall	(107, 108)
p53 agonist	Quinacrine	Stasis	No change	↓ Thrombus weight, intrathrombotic collagen area, IL-6 levels in the thrombi; promoted macrophage polarization toward an M2-like phenotype	(75)
Lipid-lowering drugs	Atorvastatin or rosuvastatin	Stasis or ferric chloride-induced	Decrease	↓ Thrombus mass, venous wall injury, platelet aggregation, clot stability, thrombus PAI-1, TF, plasma TAT complexes, inflammation markers, collagen and vein wall thickness	(109)
Nur77 agonist	Cytosporone B	Stenosis	No change	↑ Ly6C^hi^ to Ly6C^neg^ shift in the blood and splenic monocyte, accelerating intrathrombic monocyte differentiation; ↓ clot growth	(45)
Resolvin D4	Proresolving mediators	Stenosis	Decrease	↓ Neutrophil and macrophage recruitment; ↑ proresolving monocytes in the thrombus, the percentage of cells in an early apoptosis state	(110)
Impaired thrombus resolution					
MMP-2/9 inhibitor	MMP-2/9 inhibitor	Stasis	Not determined	↑ Thrombus mass and collagen content; ↓ recovery of blood flow	(63)
Synthetic fibrin-derived Bβ_15-42_ peptide	FX06	Stenosis	Decrease	↓ Microvessels and uPA expression in the thrombus; ↓ monocyte transmigration through the endothelial cell monolayer	(111)
Metabolite of 17β-estradiol	2ME	Stenosis	Decrease	↑ Thrombus weight; ↓ vein recanalization and neutrophil content, HIF1α, VEGF, and PLGF levels	(56, 112)
Tyrosine kinase inhibitor	Axitinib	Stenosis	Decrease	↑ Thrombus volume; ↓ neovascularization, collagen content	(112)
Antidepressant	Imipramine	Stenosis	No change	↑ Thrombus length and diameter; ↑ NET burden in the thrombus; ↓ macropinocytosis	(49)

↑, increase or enhance; ↓, decrease or inhibit.

APTT, activated partial thromboplastin time; DVT, deep vein thrombosis; FX-06, fibrin-derived peptide Bβ_15-42_; HIF1α, hypoxia-inducible factor 1α; MCP1/CCL2, monocyte chemotactic protein 1; 2ME, 2-methoxyestradiol; MIP-1α, macrophage inflammatory protein-1α; Mo/MΦ, monocytes/macrophages; MPO, myeloperoxidase; NETs, neutrophil extracellular traps; PAI-1, plasminogen activator inhibitor-1; PHD, prolyl hydroxylase domain; PLGF, placental growth factor; PMN, polymorphonuclear leukocyte; PT, prothrombin time; rhG-CSF, recombinant human granulocyte colony-stimulating factor; rhIL-8, recombinant human IL-8; rIL-6, recombinant murine IL-6; SMA, smooth muscle actin; TAT, thrombin-antithrombin; TF, tissue factor; uPA, urokinase-type plasminogen activator; VEGF, vascular endothelial growth factor; VEGFR, VEGF receptor; WT, wild type.

### Potential candidate drugs promoting thrombus resolution

#### Recombinant TNF-α, anti-TNF-α mAb, or etanercept

Recombinant TNF-α was found to improve thrombus resolution and accelerate blood flow recovery without affecting coagulation functions (prothrombin time and activated partial thromboplastin time). The anti-TNF-α antibody or etanercept had the opposite effect on thrombus resolution (64). However, neither TNF-α treatment nor inhibiting TNF-α with anti-TNF-α mAb or etanercept affected macrophage infiltration in the vein walls (64). Large-scale clinical trials must be conducted to verify its effectiveness and safety.

#### Recombinant IL-6 and anti-IL-6 antibody

IL-6 was shown to be mainly expressed by intrathrombotic macrophages (59). The anti-IL-6 antibody inhibited thrombus resolution, while recombinant murine IL-6 promoted thrombus resolution and accelerated blood flow recovery without affecting PT and APTT. Thus, IL-6 might have therapeutic potential in promoting thrombus resolution without affecting coagulation activities (59). However, excessive or long-term administration of IL-6 might cause various pathological disorders. Therefore, the dosage and withdrawal time of IL-6 treatment for patients with VT need large-scale clinical trials.

#### Recombinant IL-8

IL-8 was preliminarily identified as a chemotactic for neutrophils involved in acute inflammation and then discovered also to be chemotactic for endothelial cells playing a critical role in angiogenesis (113). Administration of recombinant human IL-8 (rhIL-8) was reported to enhance thrombus resolution possibly via neovascularity and inflammation (100). However, intrathrombotic macrophage content has not been detected. Therefore, the effect of IL-8 on macrophage content and function during thrombus resolution remains unclear.

#### Recombinant monocyte chemotactic protein 1

MCP1/CCL2, a member of the C-C chemokine family, is a potent chemotactic factor that regulates the migration and infiltration of monocytes/macrophages (114, 115).

It was demonstrated that thrombus MCP1 levels were elevated during thrombus resolution and recombinant rat MCP1 administration improved the organization and resolution of the thrombus possibly through chemotaxis and recruitment of monocytes into the vessel wall (101). However, another study showed that recombinant MCP1 promoted thrombus resolution and increased thrombus recanalization, without affecting macrophage recruitment (34). Therefore, whether MCP1 promoting thrombus resolution was dependent on monocyte/macrophage recruitment remains to be clarified.

#### Recombinant VEGF and VEGF receptor inhibitor

VEGF, a potent proangiogenic factor, plays a major role in vasculogenesis during the embryonic period and then in various physiological (such as the menstrual cycle, pregnancy, and wound healing and repair) and pathological angiogenesis (such as tumor growth and metastasis, macular degeneration, diabetic retinopathy, rheumatoid arthritis, myocardial ischemia, and preeclampsia) (116).

Recombinant human VEGF injected directly into the thrombus was shown to be a useful adjunct to conventional anticoagulation in dissolving VT with the increase of monocyte migration into the center of the thrombus (102). Axitinib, a tyrosine kinase inhibitor, is a potent, selective inhibitor of VEGF receptors 1, 2, and 3 (117). It was found to inhibit VT resolution with macrophage accumulation (112). Thus, when dealing with tumor patients with venous thromboembolism, the potential of antiangiogenic drugs to prolong venous occlusion should be considered.

#### Anti-factor XI antibody

Coagulation factor XI (FXI) was found to contribute to pathologic thrombus formation (118). A previous study has reported that anti-mouse FXI monoclonal antibody could reduce macrophage accumulation and accelerate the early stages of DVT resolution in mice (103). However, further studies are needed to explore how reduced FXI levels affect monocyte differentiation to macrophages and monocyte recruitment into the thrombus and whether accelerated thrombus resolution in this model is associated with the alteration in macrophage phenotype and function.

#### Anti-IFN-γ antibody

IFN-γ mainly produced by infiltrating macrophages was found to impede thrombus resolution (63). It was found that the anti-IFN-γ antibody might serve as an effective therapeutic drug for accelerating thrombus resolution without affecting coagulation function (54).

#### Recombinant human granulocyte colony-stimulating factor

Due to its ability to mobilize bone marrow cells into peripheral blood, recombinant human granulocyte colony-stimulating factor (rhG-CSF), a hematopoietic growth factor, is widely used to treat various human diseases (119). rhG-CSF was also shown to enhance VT resolution and recanalization through mobilizing mononuclear cells into the peripheral blood and promoting macrophage accumulation in thrombi (106). Therefore, rhG-CSF might be used for patients with VT, particularly for patients who are contraindicated by anticoagulation and thrombolytic therapy. However, how rhG-CSF induces macrophage accumulation in thrombi remains unclear.

#### The PAI-1 inhibitor tiplaxtinin

Oral or subcutaneous delivery of the PAI-1 inhibitor, tiplaxtinin (PAI-039), was reported to reduce thrombus weight, increase blood flow velocity, decrease both intimal thickening and fibrosis scores, and increase PMN extravasation in the vein wall of a rat stenosis model. However, PAI-1 inhibition was shown to have a non-significant decrease in monocyte extravasation in the vein wall (104).

#### The P-selectin inhibitor PSI-421

PSI-421, a small molecule inhibitor of P-selectin, was found to have greater percent vein reopening and less vein wall inflammation in a baboon model of stasis-induced DVT (105). However, it was unclear whether PSI-421 inhibits the inflammatory response of the vein wall through regulating macrophage infiltration or function.

#### The prolyl hydroxylase domain inhibitor L-mimosine

L-mimosine, an iron chelator and a prolyl hydroxylase domain (PHD) inhibitor, is also a hypoxia mimetic agent and used to increase the levels of hypoxia-inducible factor 1α (HIF1α) and induce angiogenesis both *in vitro* and *in vivo* (120, 121). L-mimosine upregulating HIF1α expression could be used to enhance thrombus resolution and recanalization which might be related to inflammatory cells including macrophages and neutrophils entering the thrombus via the vein wall (107, 108). However, the selective pan PHD inhibitors, AKB-4924 and JNJ-42041935, were found to increase intrathrombotic neovascularization without affecting thrombus resolution and macrophage accumulation in the thrombus (122). Therefore, the effect of PHD inhibitors on thrombus resolution still needs further clarification.

#### The P53 agonist quinacrine

Quinacrine, as an antimalarial drug, has been used for tapeworm infections, giardiasis, lupus erythematosus, intrauterine sterilization, Creutzfeldt-Jakob disease, and cancer (123). Quinacrine was also found to inhibit RNA virus replication and may be useful as an adjuvant antiviral compound against severe acute respiratory syndrome coronavirus-2 (SARS-CoV-2) infection (124). Recently, quinacrine was shown to enhance venous thrombus resolution in formed thrombi through altering macrophage polarization and fibrosis (75). Therefore, the short-term use of quinacrine for patients with DVT to minimize the side effects of anticoagulants may provide a clinically feasible option.

#### The Nur77 agonist cytosporone B

Cytosporone B, a Nur77 agonist, was shown to repress clot growth and promote resolution because it could enforce monocyte conversion in blood as well as accelerate intrathrombic monocyte differentiation (45). Thus, Nur77 agonists might be ideal candidates for therapeutic intervention in inflammatory monocyte activities of patients with DVT to avoid thrombus growth and speed up the resolution.

#### Resolvin D4

Resolvin D4 (RvD4), a specialized proresolving mediator, was derived from essential polyunsaturated fatty acid and enriched at the natural onset of thrombus resolution (110, 125). It was shown that repetitive delivery of resolvin D4 reduced the thrombus size and enhanced thrombus resolution through reducing neutrophil and macrophage recruitment, elevating more proresolving monocytes in the thrombus, and increasing the percentage of cells in an early apoptosis state in mice on day 8 after IVC stenosis induction (110).

### Potential candidate drugs attenuating thrombus resolution

#### The lipid-lowering drug statins

Statins, 3-hydoxy-3-methyl-glutaryl coenzyme A inhibitors, not only reduce cholesterol and cardiovascular risk but also exhibit pleiotropic effects independent of their lipid-lowering properties (126). It was demonstrated that statins could improve the resolution of established VT probably through promoting profibrinolysis, anticoagulation, antiplatelet, and antivein wall injury and reducing macrophage levels (109). Therefore, statins may be a viable therapeutic strategy to improve DVT resolution, especially in patients who cannot receive anticoagulant therapy.

#### MMP-9 inhibitor

MMP-9, a zinc-dependent endopeptidase, is one of the most complex forms of MMPs, which belongs to the gelatinase family. MMP-9 is capable of degrading extracellular matrix components (127). MMP-9 was found to be expressed in intrathrombotic macrophages (54). MMP-2/9 inhibitors impaired thrombus resolution without affecting VEGF expression (54). Thus, it is worthy of further study whether the suppressing effect of MMP-9 inhibitor on thrombus resolution is related to intrathrombotic macrophage.

#### Synthetic fibrin-derived Bβ_15-42_ peptide

The fibrin fragment Bβ_15-42_ (FX06), a naturally occurring highly charged 28 AA peptide, competes with E-fragments which were fibrin degradation products after digestion by plasmin to bind to vascular endothelial cadherin. Bβ_15-42_ was found to play a role in myocardium, kidney, and liver ischemia–reperfusion injury and severe COVID-19-associated acute respiratory distress syndrome (ARDS), owing to its anti-inflammatory properties and ability to protect the endothelial cell barrier (128).

The peptide Bβ_15-42_ was found to attenuate thrombus resolution probably through impeding monocyte endothelial transmigration, reducing macrophage numbers, microvessels, and uPA expression in the thrombus (111). Moreover, high levels of the fibrin fragment Bβ_15-42_ were found in the red thrombus and plasma of patients with CTEPH (129). Therefore, fibrin fragments suppressing thrombolysis should be taken seriously in the treatment of CTEPH.

#### 2-Methoxyestradiol

2-Methoxyestradiol (2ME), a natural metabolite of 17β-estradiol, is a potent antitumor and antiangiogenic compound (130). It was found that 2ME attenuated venous thrombus resolution by inhibiting the angiogenic response to thrombosis in the surrounding vein, accompanied by a decrease in the content of macrophages and neutrophils and the levels of HIF1α, VEGF, and PLGF (56, 112). Therefore, the potential prothrombotic effect of 2ME ought to be contemplated while treating cancer patients with venous thromboembolism.

#### Imipramine

Imipramine, a classic tricyclic antidepressant that inhibits the reuptake of norepinephrine and serotonin, is currently undergoing clinical trial and animal experiments to evaluate its anti-invasive and antimetastatic effects in the treatment of localized colorectal cancer, triple-negative breast cancer, and oral squamous cell carcinoma (131, 132). It was reported that imipramine increased NET burden in the thrombi by inhibiting macropinocytosis rather than reducing the number of macrophages infiltrating the thrombus, leading to impaired thrombus resolution 7 days after IVC ligation (49).

## Conclusion and future perspectives

Monocytes/macrophages infiltrating into the thrombus are the major effector cells at the middle and late stages of thrombus resolution. Polarization into M2 macrophages may play a more important role during thrombus resolution. They express and release various proangiogenic factors and profiber and collagen lytic enzymes, which play a role in promoting neovascularization and fibrin and collagen degradation during thrombus resolution. Moreover, multiple factors using gene deletion were demonstrated to promote or impair thrombus resolution, and their effects were associated with monocytes/macrophages in the thrombus and the adjacent vein walls. However, the function of intrathrombotic monocytes/macrophages during thrombus resolution remains to be fully elucidated. So far, some potential candidate drugs linked to monocytes/macrophages have been found to promote or impair thrombus resolution, providing an alternative therapeutic strategy for patients with DVT. However, there is still a long way to go before these candidates are translated into clinical applications.

## Author contributions

M-JL: Writing – original draft, Data curation. J-QZ: Writing – review & editing, Visualization. Z-YN: Conceptualization, Writing – review & editing. T-HY: Writing – review & editing, Visualization. Y-BC: Software, Writing – review & editing. L-CZ: Writing – review & editing, Project administration, Funding acquisition. LL: Conceptualization, Writing – original draft, Supervision, Project administration, Funding acquisition.

## Glossary

**Table:**

Arg-1	arginase 1
APTT	activated partial thromboplastin time
ARDS	acute respiratory distress syndrome
bFGF	basic fibroblast growth factor
CCR2	C-C chemokine receptor 2
Cit-H3	citrullinated histones
CTEPH	chronic thromboembolic pulmonary hypertension
DAMPs	danger-associated molecular patterns
DVT	deep vein thrombosis
ECM	extracellular matrix
FXI	coagulation factor XI
HIF1α	hypoxia-inducible factor 1α
iNOS	inducible nitric oxide synthase
IVC	inferior vena cava
IFN-γ	interferon-γ
IL-1β	interleukin-1β
IL-6	interleukin-6
IL-8	interleukin-8
M1-like	proinflammatory phenotype macrophage
M2-like	anti-inflammatory phenotype macrophage
MCP1/CCL2	monocyte chemotactic protein 1/C-C chemokine ligand 2
METs	monocyte/macrophage extracellular traps
MIP-1α/CCL3	macrophage inflammatory protein-1alpha
MMP-2	matrix metalloproteinase-2
MMP-9	matrix metalloproteinase-9
Mo/MΦ	monocyte/macrophage
NETs	neutrophil extracellular traps
2ME	2-methoxyestradiol
PAMPs	pathogen-associated molecular patterns
PAI-1	plasminogen activator inhibitor-1
PECAM-1	platelet endothelial cell adhesion molecule 1
PHD	prolyl hydroxylase domain
PLGF	placental growth factor
PMNs	polymorphonuclear leukocytes
PE	pulmonary embolism
PT	prothrombin time
PTS	post-thrombotic syndrome
rhG-CSF	recombinant human granulocyte colony-stimulating factor
rIL-6	recombinant murine IL-6
SMA	smooth muscle actin
TLR4	Toll-like receptor 4
TLR9	Toll-like receptor 9
tPA	tissue-type plasminogen activator
VEGF	vascular endothelial growth factor
VEGFR-2/KDR/Flk-1	vascular endothelial growth factor receptor 2/kinase insert domain protein receptor
VTE	venous thromboembolism
uPA	urokinase-type plasminogen activator
